# Evaluation of qualitative and quantitative taste alterations in COVID-19

**DOI:** 10.17305/bjbms.2022.6973

**Published:** 2023-03-16

**Authors:** Angela Pia Cazzolla, Roberto Lovero, Francesca Spirito, Michele Di Cosola, Luigi Santacroce, Eleonora Lo Muzio, Domenico Ciavarella, Mario Dioguardi, Vito Crincoli, Maria Pepe, Lucia Varraso, Renato Contino, Francesca Di Serio, Lorenzo Lo Muzio

**Affiliations:** 1 Department of Clinical and Experimental Medicine, Università degli Studi di Foggia, Foggia, Italy; 2 Clinical Pathology Unit, AOU Policlinico Consorziale di Bari - Ospedale Giovanni XXIII, Bari, Italy; 3 Ionian Department (DJSGEM), Microbiology and Virology Lab, Università Degli Studi di Bari, Bari, Italy; 4 Department of Translational Medicine and for Romagna, School of Orthodontics, University of Ferrara, Ferrara, Italy; 5 Department of Basic Medical Sciences, Neurosciences and Sensory Organs, ”Aldo Moro” University of Bari, Bari, Italy; 6 Transfusional Medicine Unit, University-Policlinico, Bari, Italy; 7 Consorzio Interuniversitario Nazionale per la Bio-Oncologia (C.I.N.B.O), Chieti, Italy

**Keywords:** Coronavirus disease 2019 (COVID-19), taste dysfunction, dysgeusia

## Abstract

A large percentage of coronavirus disease 2019 (COVID-19) patients have taste dysfunction. Interleukin 6 (IL-6) levels in mild and moderate COVID-19 patients with the type (quantitative or qualitative) of taste disorders were compared in this observational study. The 208 COVID-19 patients (118 men and 90 women) revealing only taste dysfunctions as prodromic symptoms were classified as mild and moderate patients. Survey results were used to evaluate the taste disorder. The IL-6 levels were measured using a chemiluminescence assay. Statistical analysis was conducted using the Wilcoxon rank, Welch’s, and Mann–Whitney tests. The findings revealed that neither the presence of dysgeusia or phantogeusia nor the perception of sour and salty, differed significantly between moderate and mild patients (*P* > 0.05). But between moderate and mild patients, there were significant differences in umami, bitter, sweet, and parageusia perception (*P* < 0.05). There was an impairment of multiple tastes up to ageusia in patients with high IL-6 levels. The findings demonstrated that parageusia and dysfunctions in umami, bitter, and sweet taste perception can be indicators of more severe forms of COVID-19.

## Introduction

Many coronavirus disease 2019 (COVID-19) patients experience taste dysfunctions and self-reported taste loss may have a more significant prognostic significance than other COVID-19 symptoms like fatigue, fever, or cough [1]. According to some studies, the prevalence of taste disturbances in mild-to-moderate coronavirus disease ranges from 71% to 88.8% (78.9% for hypogeusia/ageusia and 21.1% for parageusia) (COVID-19). Two meta-analyses revealed a combined gustatory dysfunction of 38.2%–49.0% [2–5].

Taste disturbances are divided into qualitative and quantitative categories. Dysgeusia (a distortion of taste perception elicited by previously acceptable foods that become unpleasant), parageusia (an altered taste perception, more often unpleasant with external stimulus), and phantogeusia are examples of qualitative disturbances (the perception of metallic or salty taste without external stimulus). Quantitative disturbances include ageusia (total deficit), hypogeusia (loss of certain tastes), and hypergeusia (the increase of gustatory sensitivity) [6].

Taste perception and smell perception are closely related, and salivation, a healthy neuronal network, and the right stimulation of taste buds are all necessary for optimal taste perception. Taste buds are chemoreceptors present in the fungiform, foliate, and circumvallate papillae of the dorsal mucosa of the tongue but also in the palate, pharynx, and larynx. Trigeminal nerve endings, which convey information about temperature, texture, and pain, are found in filiform papillae [7].

To maintain taste bud homeostasis and intact taste function, progenitor cells in the surrounding tissues must continuously differentiate into the specific types of taste cells, with the average lifespan of taste cells estimated to be between 8 and 12 days [8].

Each taste bud is composed of 150–300 epithelial cylindrical cells and contains 5 types of cells: type 1, 2, 3 cells (specialized epithelial cells for recognizing taste), type 4 cells, and supporting cells and neuronal processes [9]. The type 1 cells, which are glial-like cells and have several long microvilli, are thought to be salty taste-mediating cells. The type 2 cells contain the G-protein-coupled receptors (GPCRs) and are thought to transduce sweet, umami, and bitter tastes. The type 3 cells are presynaptic cells that mediate communication from the type 2 cells via P2Y adenosine receptors, transduce salty and sour taste [10], and mediate signals to the afferent neurons via serotonin release. The type 4 cells are basal precursor cells and differentiate into type 1, 2, and 3 taste cells during rapid cell turnover in taste buds [8].

To date, the mechanisms of taste reception are not well understood. Even though it has been proposed that the epithelial sodium channel (ENAC) (amiloride-sensitive ion channels) and add sensing ion channel (ASIC) are involved in the perception of salt in rodents, the receptor system for salty taste has not yet been identified [11]. Two members of the transient receptor potential superfamily of ion channels, as well as ASIC, which are found on various populations of papillae, mediate the sour taste. Other tastes are perceived by GPCRs, which are members of the C class receptors [12].

Artificial sweeteners and sugars, in particular, bind to the sweet taste receptors T1R2/3 and trigger downstream pathways (sugars activate a phospholipase C-dependent pathway, while artificial sweeteners activate the adenylyl cyclase pathway). Monosodium glutamate binds to the umami receptor, T1R1/3, and activates a phospholipase C pathway. Bitter taste activates 25 T2Rs potassium channel, and subsequently phospholipase C and adenylyl cyclase pathways [8, 12].

The transfer of information to the central nervous system (CNS) takes place via three nerves: the chords tympani, the glossopharyngeal nerve, and the trigeminal after the bond between gustatory stimuli molecules and the specific receptor [12]. Upon reaching the gustatory cortex, a gustotopic map is present [13].

Severe acute respiratory syndrome coronavirus 2 (SARS-CoV-2) entry is mediated by angiotensin-converting enzyme (ACE) receptors [14–18] and TMPRSS2 proteases [19]. TMPRSS2 proteases are expressed in the mesenchyme and epithelium of the soft palate as well as the epithelium of the tongue in mice. They demonstrated a progressive increase over developmental stages (mice and humans share similar gene expression patterns for ACE receptor and TMPRSS2 proteases) [20]. The oral tongue’s keratinocytes express more ACE2 receptors than do other buccal and gingival tissues, and lymphocytes within oral mucosa [15]. In particular, ACE2 receptors are not expressed in taste buds’ specialized cells, progenitor cells that surround taste buds, or taste buds’ epithelial cells. A small subpopulation of epithelial cells in the basal region of filiform non-taste buds and a small proportion of type 3 taste cells contain ACE receptors. Therefore, the involvement of the epithelial cells of the non-gustatory papilla rather than direct infection of taste cells with SARS-CoV-2 is what causes the loss of taste [20].

Additionally, signal interactions between the spike protein and the ACE2 receptor on olfactory mucosal cells have shown that SARS-CoV-2 is neuroinvasive and neurotropic [21]. This specific tropism results in an infection of the CNS and concurrent neuronal loss [21].

There is no consensus on the possible causes of taste loss. It may be caused by a variety of factors, including neurologic damage [22], tissue hypoxia [23], binding of SARS-CoV-2 to sialic acid receptors, and interfering with the transport of taste substances by glycoproteins before they can be detected [24], changes in cellular zinc homeostasis of gustatory cells for zinc [25], local and/or systemic immune responses [26, 27], inflammatory reaction [28] with cellular changes that could alter taste [29]. There is an inflammatory storm caused by the release of 150 different inflammatory cytokines and chemical mediators during SARS-CoV 2 infection from both immune and non-immune cells (cytokine release syndrome) [30, 31].

Interleukin 6 (IL-6) is known to play a major role in cytokine release syndrom and is known to cause the release of several acute-phase proteins, including (C-reactive protein, fibrinogen, C3, C4, etc.). It is also known to be a poor prognostic indicator for SARS-CoV-2 infection. Elevated levels of IL-6 were significantly associated with severe clinial manifestations affecting the respiratory tract, heart, kidneys, liver, and gastrointestinal tract with microbiota dysbiosis [32], spleen, central nervous system, lymph nodes, and skin.

This observational study’s objective aimed to compare the IL-6 levels between mild and moderate COVID-19 patients according to the kind (quantitative or qualitative) of taste disorders. To identify which taste disorders are most compromised in mild and moderate COVID-19 patients, the levels of IL-6 were also compared with the number of food flavors that are typically present at home (household tastes). This was done to determine whether the alteration of a particular taste is a sign of more severe clinical manifestations.

## Materials and methods

This study was conducted from 15 March 2020 to 15 April 2021. The study included patients with mild-to-moderate COVID-19. Table 1 lists inclusion and exclusion criteria.

**Table 1:** Inclusion and exclusion criteria

**Inclusion criteria**	**Exclusion criteria**
- Age > 18 years	- Patients without a laboratory-confirmed diagnosis of COVID-19 infection
- Laboratory-confirmed COVID-19 infection (reverse transcription-polymerase chain reaction)	- Patients with taste dysfunctions before the epidemic (congenital ageusia, side effects of drugs, previous surgery of ear and wisdom teeth, radiotherapy of the oral and pharyngeal cavities, chemotherapy, diabetes mellitus, increasing age, and institutionalization or acute hospitalization)
- Patients with only taste dysfunction as the prodromic symptom	- Nutrient deficiency
	- Insufficient hydration
	- Head trauma
	- Hypothyroidism
	- Heart disease
	- Liver diseases
	- Kidney diseases
	- Malignancies (oral and tongue carcinoma)
	- Ear infection
	- Patients with neurodegenerative and psychiatric disorders (Parkinson’s disease, major depression, and Alzheimer’s disease)
	- Upper respiratory infection
	- Allergic rhinitis, asthma
	- Oral cavity infections (related to the use of dental prostheses, candidiasis)
	- Poor oral hygiene
	- Burning mouth syndrome
	- Smoke

Clinical manifestations were used to categorize the patients into mild and moderate groups (Table 2) and a survey was used to determine whether there was any qualitative (dysgeusia, parageusia, and phantogeusia) and quantitative (ageusia, hypogeusia, and hypergeusia) taste disturbances.

**Table 2:** General characteristics, associated symptoms, and associated comorbidities of 208 COVID-19 patients

*General characteristics*	
Sex	Age (years)
Male 118 (57%)	59 ± 13
Female 90 (43%)	56 ± 16
Days from COVID-19 symptoms onset	4 ± 1
*Clinical classification*	
**MILD 127 (61%)**	54 ± 13
Male 62 (49%)	53 ± 15
Female 65 (51%)	49 ± 14
**MODERATE 81 (39%)**	62 ± 12
Male 56 (69%)	69 ± 11
Female 25 (31%)	66 ± 12
*Associated symptoms*	N (%)
Cough	176 (85)
Muscle or joint pains	194 (93)
Stuffy nose	97 (46)
Chest pain	144 (69)
Fever	196 (94)
Felt tired	153 (73)
Problems breathing	66 (80.4)
Asthenia	185 (89)
Rhinorrhea	78 (36)
Headache	102 (49)
Abdominal symptoms	29 (14)
Sore throat	131 (63)
Vomit	59 (28)
Nausea	91 (44)
Diarrhea	31 (15)
Loss of appetite	159 (76)
*Associated comorbidities*	N (%)
Gastroesophageal reflux disease	101 (48)
Hypertension	99 (47)
Thyroid diseases	52 (25)
Respiratory insufficiency	29 (14)

Each patient had their venous sample drawn when they were admitted to the hospital. The venous samples were collected in 5 ml Vacutainer tubes without anticoagulants, then centrifuged (1000 × *g*, 15 min, 4 ^∘^C) and stored at −80 ^∘^C until analysis. The IL-6 levels (normal values 0–7 pg/mL) have been assessed with a chemiluminescence assay using Cobas e801 (Roche Instrumentation).

Each patient was given a survey that had two sections. The first part was for health care professionals and it included general questions (age, sex, residence, work activity, smoking habit, previous institutionalization or acute hospitalization), the presence of systemic diseased (hypertension, diabetes, gastrointestinal, thyroid, heart, kidney, liver pathologies, upper respiratory infection, and head trauma), and local diseases (oral cavity infections, candidiasis, poor oral hygiene, and burning mouth syndrome). The patients filled out the second section of the survey. It looked into the type of taste alteration and the time of onset of taste disorder. The US NHANES 2011–2014 protocol’s Taste and Smell Questionnaire Section was used as a guide to assess gustatory function (CDC 2013b) [33].

The survey examined the type of taste disturbance (salty, bitter, sour, umami, and sweet) assessing the perception of the flavors of foods normally present at the home (household tastes). By giving each household that was not perceived a score; the total number of unperceived tastes in the household was calculated. The score was calculated by assigning a value of 0 when all tastes were perceived and 5 when they were not perceived. Furthermore, it was asked if the taste of table salt that had been dissolved in the water was perceived (as salty), if the taste of sugar dissolved in the water was perceived (as sweet), if the lemon juice was perceived (as sour), if the taste of bitter coffee was perceived (bitter), and if the taste of parmesan, a food that contains glutamate, was perceived (umami). It was asked whether the patients had unpleasant tastes (metallic or salty) with or without food to assess the presence of qualitative disturbances.

### Ethical statement

The study was performed according to the guidelines of the Declaration of Helsinki and approved by the Ethics Committee of Bari (Italy) N. 6388 COVID19 DOM protocol number 0034687/12-05-2020. All participants in the study provided their informed consent.

### Statistical analysis

MedCalc software was used for all analyses. Age, sex, symptoms associated, and taste disorders are reported in numerals and percentages of the total. Using descriptive statistics for quantitative variables, the mean ± SD is provided. The Kolmogorov–Smirnov test and Shapiro–Wilk test were used to determine whether the distribution of the variables was normal. A non-normal distribution of variables of the variables was demonstrated by both tests.

Both mild and moderate patients with taste dysfunction and IL-6 values in relation to the type of reported taste disorder were assessed using the Wilcoxon rank test, Welch’s t-test, or unequal variance t-test (quantitative or qualitative disorders). The differences between independent groups of mild and moderate patients as well as the various taste alterations were assessed using the Mann–Whitney test. A 5% *P* value threshold was adopted for all tests used.

## Results

In total, 208 COVID-19 patients having taste disturbances (118 [57%] men aged 59 ± 13 and 90 [43%] women aged 56 ± 12 years) were selected.

Out of 1155 COVID-19 patients, 821 (71%) presented chemosensitive dysfunctions. Of them, 208 (25%) patients (118 men aged 59 ± 13 and 90 women aged 56 ± 12 years) showed only taste dysfunctions as prodromic symptoms, 286 (35%) revealed only smell dysfunctions as prodromic symptoms, and 180 (22%) presented with smell and taste disorders. One hundred forty-seven (18%) patients were excluded because 37 (25%) gave no response, 35 (24%) required intensive care admission (critical patients), 13 (9%) presented with allergic rhinitis, 4 (3%) finished chemotherapy, 2 (1%) radiotherapy for oral and oropharyngeal cancer recently, 15 (10%) presented with diabetes, 15 (10%) were affected by liver diseases, 10 (7%) affected by kidney diseases, 9 (6%) affected by hypothyroidism, and 7 (5%) affected by neurologic and psychiatric diseases.

**Figure 1.**
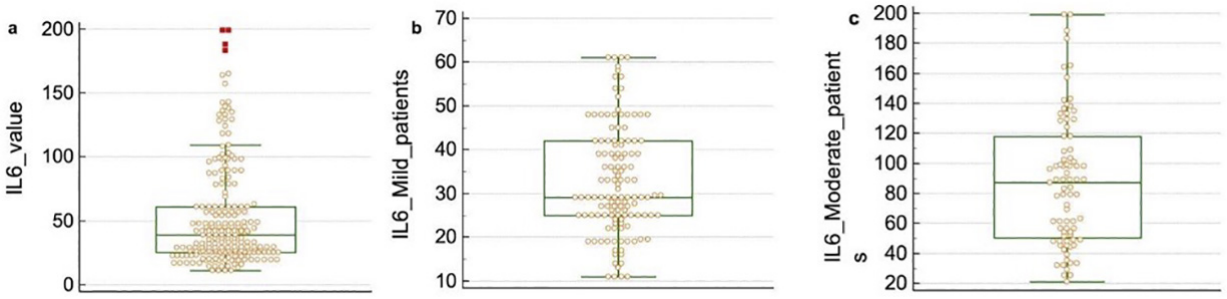
**Distribution of IL-6 values.** (A) In total sample, (B) in mild patients, and (C) in moderate patients. IL-6: Interleukin 6.

When IL-6 levels were compared between patients with mild and moderate disease, those with a severe clinical course had higher IL-6 values (Figure 1).

The Wilcoxon rank test displayed a statistically significant difference in IL-6 levels in moderate vs mild patients with ageusia, hypogeusia (score), and parageusia (*p* < 0.05). Patients with hypogeusia were divided into groups: those who reported deficits in the perception of acid and/or salty, mediated by ion receptors, and patients that reported a deficit in the perception of sweet and/or bitter and/or umami, mediated by GPCRs.

There were statistically significant differences (*P* < 0.05) in IL-6 values between moderate vs mild patients for quantitative umami, bitter, and sweet taste disturbances (GPCRs). There were no significant differences (*P* > 0.05) in IL-6 values between moderate vs mild patients for quantitative sour and salty taste disturbance (ion receptors) (Table 3).

**Table 3:** Assessment of IL-6 values in moderate vs mild patients with qualitative and quantitative taste disorders

**Parameters**	***P* value**
IL-6 value in moderate vs mild patients	< 0.05
*Taste quantitative disorders*	
IL-6 value in moderate vs mild patients (ageusia)	< 0.05
IL-6 value in moderate vs mild patients (hypogeusia-score)	< 0.05
*Taste quantitative disorders: hypogeusia*	
IL-6 value in moderate vs mild patients (sour and salty)	> 0.05
IL-6 value in moderate vs mild patients (umami, bitter, and sweet)	< 0.05
*Taste qualitative disorders*	
IL-6 value in moderate vs mild patients (parageusia)	< 0.05
IL-6 value in moderate vs mild patients (dysgeusia)	> 0.05
IL-6 value in moderate vs mild patients (phantogeusia)	> 0.05

IL-6: Interleukin 6.

There was no significant difference in IL-6 values between moderate and mild patients with dysgeusia and phantogeusia (*P* > 0.05) (Table 3).

Welch’s t-test revealed a statistically significant difference between the mean values of IL-6 in moderate (67.8 pg/mL) vs mild (22.9 pg/mL) patients and between patients affected by hypogeusia and parageusia (*P* < 0.05) and in patients with an altered perception of umami, bitter, and sweet (*P* < 0.05). Patients with dysgeusia, phantogeusia, and an altered sense of sour and salty who were moderate vs mildly affected by IL-6 did not differ significantly from each other (*P* > 0.05) (Table 4).

**Table 4:** Assessment of mean values IL-6 in moderate vs mild patients with taste disorders (Welch’s t-test) and assessment in moderate vs mild patients with ageusia, parageusia, dysgeusia, phantogeusia, sour, salty, umami, bitter, and sweet disorders (Mann–Whitney’s test)

**Parameters**	***P* value**
IL-6 value in moderate vs mild patients	< 0.05
*Taste quantitative disorders*	
IL-6 value in moderate vs mild patients (ageusia)	< 0.05
IL-6 value in moderate vs mild patients (hypogeusia-score)	< 0.05
*Taste qualitative disorders*	
IL-6 value in moderate vs mild patients (parageusia)	< 0.05
IL-6 value in moderate vs mild patients (dysgeusia)	> 0.05
IL-6 value in moderate vs mild patients (phantogeusia)	> 0.05
*Taste quantitative disorder (hypogeusia)*	
IL-6 value in moderate vs mild patients with hypogeusia (sour and salty)	> 0.05
IL-6 value in moderate vs mild patients with hypogeusia (umami, bitter, and sweet)	< 0.05
**Mann–Whitney test**	
**Parameters**	***P* value**
*Taste quantitative disorders*	
Moderate vs mild patients (ageusia)	< 0.05
Moderate vs mild patients (hypogeusia-score)	< 0.05
*Taste qualitative disorders*	
Moderate vs mild patients (parageusia)	< 0.05
Moderate vs mild patients (dysgeusia)	> 0.05
Moderate vs mild patients (phantogeusia)	> 0.05
*Taste quantitative disorder (hypogeusia)*	
Severe vs mild patients (sour and salty)	< 0.05
Severe vs mild patients (umami, bitter, and sweet)	> 0.05

IL-6: Interleukin 6.

Wilcoxon rank test and Welch’s test showed that IL-6 could play a key role in the onset of quantitative (ageusia and hypogeusia) and qualitative (parageusia) taste disorders in COVID-19 patients.

Mann–Whitney’s test was utilized to compare moderate vs mild patients affected by taste disorders. There were significant differences in the presence of ageusia, hypogeusia (score), parageusia, and the perception of umami, bitter, and sweet (*P* < 0.05). Ageusia, hypogeusia (score), parageusia, and the perception of umami, bitter, and sweet all showed significant differences (*P* > 0.05). Mann–Whitney’s test revealed that tastes perceived through GPCRs (umami, bitter, and sweet) are more impaired in moderate COVID-19 patients with taste alterations than in mild ones (Table 4).

## Discussion

In COVID-19 patients, taste changes that are accompanied by odor disorders are prodromal symptoms and play a crucial role in the early diagnosis of the condition [34–36].

This is the first observational study that analyzed IL-6 levels in COVID-19 patients with only taste dysfunction.

The findings of the current study confirmed the literature data showing a statistically significant relationship between the increased levels of IL-6 in moderate vs mild COVID-19 patients and taste disorders [27].

They demonstrated that in moderate patients with high IL-6 levels an impairment of multiple tastes up to ageusia (*P* < 0.05) was present. They also revealed that in mild patients with milder inflammatory clinical manifestations sour and salty tastes mediated by ion receptors were impaired (*P* > 0.05), while umami, bitter, and sweet tastes perceived through GPCRs were present in patients with more severe clinical manifestations (*P* < 0.05). In terms of qualitative disorders, phantogeusia and dysgeusia were present in moderate patients (*P* < 0.05) while phantogeusia and dysgeusia were present in mild patients (*P* > 0.05).

These data indicated that following the entry of SARS-CoV-2 into the cells of the non-gustatory filiform papillae where there are ACE receptors, an inflammatory process is produced with the release of cytokines that act on taste cells. In fact, Patra et al. [37] demonstrated that the interaction of spike protein with the ACE receptors triggers several molecular processes in the infected cells that result in the production of IL-6 and other proinflammatory cytokines.

Choudhury et al. [38] suggested that the inflammatory response could be due to interaction between Toll-like receptors, particularly Toll-like receptor 4, present on type 2 cells that express gustducin [28] and virus with tissue damage [39] Viral pathogens in the oral cavity can stimulate Toll-like receptor 4 and cause inflammatory cytokines to be produced by taste buds, which may affect both normal taste transduction and taste dysfunction caused by taste cell turnover.

The inflammatory response could first damage the cells responsible for the sour and salty taste and then the cells responsible for the sweet, umami, and bitter taste. A condition known as ageusia could develop if the inflammatory process continues.

In mild patients, amiloride-sensitive ion channels ENAC and ASIC for salty taste and ion channels PKD1L3, PKD2L1, and ASIC for sour taste were frequently impaired, whereas, in moderate patients, both ionic channels and GPCRSs were impaired.

The ion channels can be affected by cytokines, chemokines, and other inflammatory mediators. The relationship between inflammation and ion transport has been shown in various studies: inflammatory mediators may alter epithelial ion transport with impaired cell permeability and may influence cell proliferation with the emergence of less differentiated cells with fewer ion channels. The transmission of sour and salty tastes to the CNS may change as a result of these changes [40].

Furthermore, the role of cytokines has been shown in the alterated transmission of sweet, umami, and bitter tastes to the CNS by determining dysfunction and desensitization of C class receptors (GPCRs) [41].

**Figure 2.**
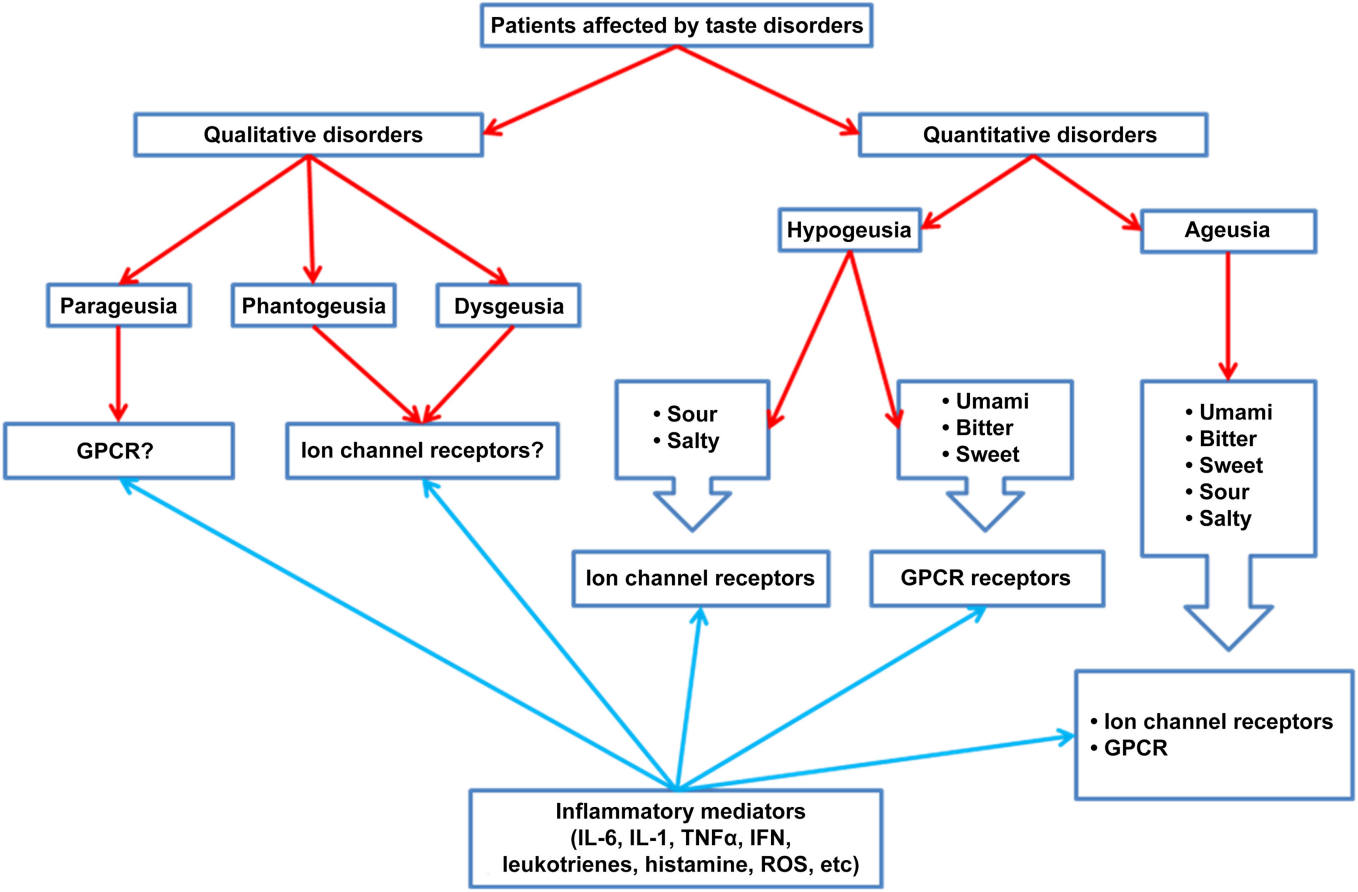
**Summary scheme of the probable mechanisms of onset of taste disorders (qualitative and quantitative).** GPCR: G-protein-coupled receptors; IL: Interleukin; INF: Interferon; TNF-α: Tumor necrosis factor α; ROS: Reactive oxygen species.

In the context of qualitative disorders, both dysgeusia and phantogeusia are present in mild and moderate forms while parageusia is present in moderate forms. There have not been many studies done on the pathophysiological underpinnings of the onset of these disorders. The findings suggest that dysgeusia and phantogeusia may be related to ion receptor dysfunction [42], whereas parageusia may be related to greater involvement of GPCRs (Figure 2).

Additionally, there is currently no approved treatment for the improvement of taste alterations in COVID-19 patients. To alleviate the symptoms of anosmia and dysgeusia, corticosteroids have been suggested as a local therapy in the form of steroid nasal spray or steroid paste [34]. In fact, zinc deficiency is frequently observed in COVID-19 patients, and given that zinc plays a role in taste function, oral zinc supplementation may reduce the symptoms of dysgeusia by facilitating the transfer of information from taste cells to gustatory nerve fibers [35].

The limitations of the present study include the limited number of patients included in studied sample and absence of cooperation of the interviewed patients due to the clinical manifestations. Also, only a questionnaire with a subjective and non-objective evaluation was used to assess taste disorders. The fact that this is an observational study, with the goal of generating hypotheses that may or may not be confirmed by follow-up, properly designed experimental studies, is another limitation.

## Conclusion

According to this study, moderate patients exhibit more impairment in their ability to taste (umami, bitter, and sweet) through GPCRs than mild patients. It also demonstrated that parageusia-related taste dysfunctions in the areas of sweetness, bitterness, and umami may be indicators of more severe COVID-19 forms. As a result, our study demonstrates how the type of taste alteration, which is correlated to the levels of inflammatory mediators such as IL-6, may serve as a prognostic indicator of the course of COVID-19.

Additionally, it offered fresh avenues for investigation into the physiopathology of taste disorders. To verify the results, experimental studies on broader statistical data are required.
